# Achieved blood pressure post-acute kidney injury and risk of adverse outcomes after AKI: A prospective parallel cohort study

**DOI:** 10.1186/s12882-021-02480-1

**Published:** 2021-07-29

**Authors:** Ian McCoy, Sandeep Brar, Kathleen D. Liu, Alan S. Go, Raymond K. Hsu, Vernon M. Chinchilli, Steven G. Coca, Amit X. Garg, Jonathan Himmelfarb, T. Alp Ikizler, James Kaufman, Paul L. Kimmel, Julie B. Lewis, Chirag R. Parikh, Edward D. Siew, Lorraine B. Ware, Hui Zeng, Chi-yuan Hsu, for the Assessment, Serial Evaluation, and Subsequent Sequelae in Acute Kidney Injury (ASSESS-AKI) study investigators

**Affiliations:** 1 Division of Nephrology, University of California, San Francisco School of Medicine, San Francisco, CA USA; 2 Department of Epidemiology and Biostatistics, University of California, San Francisco, CA USA; 3 Division of Nephrology, University of British Columbia, Vancouver, BC Canada; 4 Departments of Medicine and Anesthesia, University of California, San Francisco, CA USA; 5 Division of Research, Kaiser Permanente Northern California, Oakland, CA USA; 6 Department of Public Health Sciences, Pennsylvania State University College of Medicine, Hershey, PA USA; 7 Division of Nephrology, Icahn School of Medicine at Mount Sinai, New York, NY USA; 8 Department of Medicine, Epidemiology and Biostatistics, Western University, London, Ontario Canada; 9 Division of Nephrology, University of Washington, Seattle, WA USA; 10 Division of Nephrology & Hypertension and Vanderbilt Center for Kidney Disease, Vanderbilt University Medical Center, Nashville, TN USA; 11 Renal Section, Veterans Affairs New York Harbor Health Care System and New York University School of Medicine, NY New York, USA; 12 Division of Kidney, Urologic and Hematologic Diseases, Digestive and Kidney Diseases, National Institute of Diabetes, National Institutes of Health, Bethesda, MD USA; 13 Division of Nephrology, Johns Hopkins School of Medicine, Baltimore, MD USA; 14 Tennessee Valley Health Services Nashville Veterans Affairs Hospital, Nashville, TN USA; 15 Departments of Medicine and Pathology, Microbiology and Immunology, Vanderbilt University, Nashville, TN USA; 16 Division of Nephrology, University of California, San Francisco, 533 Parnassus Avenue, U-400, CA 94143-0532 San Francisco, USA

**Keywords:** AKI, blood pressure, hypertension

## Abstract

**Background:**

There has recently been considerable interest in better understanding how blood pressure should be managed after an episode of hospitalized AKI, but there are scant data regarding the associations between blood pressure measured after AKI and subsequent adverse outcomes. We hypothesized that among AKI survivors, higher blood pressure measured three months after hospital discharge would be associated with worse outcomes. We also hypothesized these associations between blood pressure and outcomes would be similar among those who survived non-AKI hospitalizations.

**Methods:**

We quantified how systolic blood pressure (SBP) observed three months after hospital discharge was associated with risks of subsequent hospitalized AKI, loss of kidney function, mortality, and heart failure events among 769 patients in the prospective ASSESS-AKI cohort study who had hospitalized AKI. We repeated this analysis among the 769 matched non-AKI ASSESS-AKI enrollees. We then formally tested for AKI interaction in the full cohort of 1538 patients to determine if these associations differed among those who did and did not experience AKI during the index hospitalization.

**Results:**

Among 769 patients with AKI, 42 % had subsequent AKI, 13 % had loss of kidney function, 27 % died, and 18 % had heart failure events. SBP 3 months post-hospitalization did not have a stepwise association with the risk of subsequent AKI, loss of kidney function, mortality, or heart failure events. Among the 769 without AKI, there was also no stepwise association with these risks. In formal interaction testing using the full cohort of 1538 patients, hospitalized AKI did not modify the association between post-discharge SBP and subsequent risks of adverse clinical outcomes.

**Conclusions:**

Contrary to our first hypothesis, we did not observe that higher stepwise blood pressure measured three months after hospital discharge with AKI was associated with worse outcomes. Our data were consistent with our second hypothesis that the association between blood pressure measured three months after hospital discharge and outcomes among AKI survivors is similar to that observed among those who survived non-AKI hospitalizations.

**Supplementary Information:**

The online version contains supplementary material available at 10.1186/s12882-021-02480-1.

## Background

Patients who experience hospitalized AKI are more likely to experience long-term risks of more rapid loss of kidney function measured by estimated glomerular filtration rate (eGFR), subsequent hospitalization for heart failure and all-cause mortality [1–5]. This has spurred considerable interest in better understanding how patients hospitalized with AKI should be managed after discharge. The National Institute of Diabetes and Digestive and Kidney Diseases (NIDDK) hosted a workshop dedicated to this topic in 2019 [6]. The NIDDK workshop report specifically noted that “How patients should be treated – including processes of care, level of hypertension control, and the use of blockers of the renin-angiotensin system -- in the post-AKI outpatient setting is unknown.”[7] Recently, the Caring for OutPatiEnts after Acute Kidney Injury (COPE-AKI) Funding Opportunity Announcement invited applications for studies of post-AKI interventions in randomized clinical trials, potentially including different blood pressure targets, inhibition of the renin-angiotensin-aldosterone system, use of telemedicine, and other interventions [8]. While there have been several reports on the use of blockers of the renin-angiotensin system after AKI [9–14], studies that have systematically collected data on post-AKI blood pressure (BP) level and examined associations with subsequent adverse outcomes are scarce.

Optimal BP targets may not be different after a hospitalization with AKI than after a hospitalization without AKI. Recent literature suggests that prognostic factors for adverse outcomes after AKI (such as proteinuria) [15] and risk-benefit ratio of renin-angiotensin system blockade therapy after AKI [9, 10, 13, 14] are actually similar to those seen in other high-risk patients who have been hospitalized and either have or are at high risk for having chronic kidney disease (CKD).

Motivated by the lack of data to inform the design of potential post-AKI intervention trials, we analyzed data from a prospective parallel cohort study to test two hypotheses: (i) higher achieved BP measured 3 months after an index hospitalization would associate with subsequent adverse events and (ii) these associations would not be modified by whether or not AKI occurred during that index hospitalization.

## Materials and methods

### Study population

ASsessment, Serial Evaluation, and Subsequent Sequelae in Acute Kidney Injury (ASSESS-AKI) is a parallel, matched, prospective cohort study of participants with and without AKI during hospitalization at four North American centers between December 2009 and February 2015. Details of the study have been published [1, 13, 16]. Briefly, AKI during the index hospitalization was defined as a relative increase of ≥ 50 % or absolute increase of ≥ 0.3 mg/dL in peak inpatient serum creatinine concentration (SCr) above the closest in time, outpatient, non-emergency department SCr obtained 7 to 365 days before admission. For patients from the consortium led by Yale, SCrs between 1 and 7 days before cardiac surgery were also allowed, provided the patient was undergoing elective surgery. Absence of AKI was defined as having both < 20 % relative increase and ≤ 0.2 mg/dL absolute increase in peak inpatient SCr compared with baseline outpatient SCr. Patients were initially matched on clinical center and pre-admission CKD status, with additional matching on an integrated priority score based on age, history of cardiovascular disease, presence of diabetes mellitus, category of pre-index hospitalization eGFR and treatment in an ICU [1, 13, 15]. Enrolled participants had an outpatient study visit 3 months after discharge from the index hospitalization (hereafter referred to as the baseline study visit) and follow-up in-person study visits nine months after the baseline visit and annually thereafter, with interim phone contacts at approximately 6-month intervals. Institutional review boards at the participating centers (Data Coordinating Center: Pennsylvania State University and Clinical Research Center networks: Kaiser Permanente Northern California (Oakland, CA), Vanderbilt University Medical Center (Nashville, TN), University of Washington (Seattle, WA), and Translational Research Investigating Bio-marker Endpoints in Acute Kidney Injury (TRIBE-AKI) network (New Haven, CT; Cincinnati, OH; London, Ontario; Montreal, Quebec)) approved the study, and all methods were carried out in accordance with relevant guidelines and regulations. Written informed consent was obtained from participants.

### Assessment of BP

Resting BP was measured for each participant at the 3-month post-hospitalization baseline visit by trained research personnel using an Omron automated oscillometric BP cuff. Three measurements were taken 30 s apart with the participant seated in a chair for 5 min prior to the first measurement. We performed analyses based on the mean of these three measurements.

### Outcomes

Follow-up continued until death, loss to follow-up, or study end on November 30, 2018. At each contact, the occurrence of any hospitalizations was ascertained by self-report and/or surveillance of electronic medical record systems with validation of clinical outcomes through physician adjudication of medical records as previously described [1, 13, 16]. Subsequent hospitalized AKI (hereafter referred to as subsequent AKI) was defined as a ≥50 % relative increase between the nadir and peak inpatient SCr value. Loss of kidney function was defined as ≥50 % relative decrease in eGFR from the baseline study visit or the occurrence of end stage kidney disease (ESKD) (receipt of chronic outpatient dialysis or kidney transplant). To reduce risk of misclassification from different frequencies of SCr measurement, only eGFR measurements from research study visits were used to define loss of kidney function (i.e., not including any eGFR or SCr measurements performed as part of clinical care). All-cause mortality was identified primarily through surveys of subject or proxy contacts, review of medical records and death certificates. Heart failure events were hospitalizations for clinical heart failure, identified by discharge diagnosis codes and adjudicated using Framingham Heart Study clinical criteria [17].

### Covariates

All covariates were measured at the baseline study visit including self-reported sociodemographic factors, smoking status, body mass index, medication use, and comorbidities. Blood samples were collected to measure SCr at the ASSESS-AKI Central Laboratory at the University of Minnesota using an isotope dilution mass spectrometry (IDMS)-traceable assay. Urine total protein was quantified using a turbidimetric method and urine albumin concentration using a nephelometric method in a central laboratory.

### Statistical analysis

For the 769 ASSESS-AKI participants who experienced AKI during the index hospitalization and the 769 participants who did not experience AKI, we built separate cubic polynomial spline models examining the association between systolic BP (SBP) observed 3 months after discharge and risk of subsequent AKI within the framework of proportional hazards regression models. We adjusted these models for center, age, gender, race, smoking status, history of myocardial infarction or revascularization, diabetes mellitus, heart failure, and 3-month baseline visit CKD-EPI eGFR, body mass index, urine protein:creatinine ratio, use of diuretics, blockers of the renin-angiotensin system, statins, beta blockers and calcium channel blockers. For each model, we constructed two cubic polynomial curves of the SBP that connected at the knot point of 120 mmHg. We imposed the conditions that the two splines, along with their first two derivatives, were continuous at the knot point for the sake of rendering a smooth curve. The same models were then used to examine association between SBP and risks of loss of kidney function, all-cause mortality, and hospitalization for heart failure. In addition, to illustrate an example of the relative hazards at a particular BP, we reported the model-based estimate of the hazard ratio, along with its confidence interval, for SBP 140 mmHg compared to SBP 120 mmHg. We selected 140 mmHg vs. 120 mmHg since these SBP targets might be considered for a future trial of blood pressure control among AKI survivors, and these were the SBP targets in the SPRINT trial [18] that found more AKI events in those randomized to the 120 mmHg target. For all outcomes, participants were censored for end of study, death or loss to follow-up. For the outcome of AKI after the baseline study visit, subjects were also censored if ESKD occurred first.

To formally test for interaction of AKI status during the index hospitalization on the shape of the spline curves for SBP and outcomes, we constructed interaction terms between AKI status and each component of the cubic polynomial spline in a model containing the full cohort of all 1538 patients. We then applied approximate t tests with the resultant partial maximum likelihood estimates of the interaction terms to assess their statistical significance.

In secondary analyses, we examined the mean of the three baseline visit diastolic BP (DBP) measurements with the knot point of 80 mmHg. We also examined alternative definitions for subsequent hospitalized AKI episode with (1) AKI defined as a ≥ 50 % relative increase from the most recent outpatient study visit SCr (done within one year) to the peak inpatient SCr and (2) AKI defined as a≥ 50 % relative increase or ≥ 0.3 mg/dL absolute increase from the most recent outpatient study visit SCr to the peak inpatient SCr [13].

Less than 0.5 % of data on covariates was missing. We performed a complete case analysis, excluding 27 participants with AKI during the index hospitalization and 20 participants without AKI during index hospitalization (Supplemental Figure S1).

## Results

### Cohort characteristics

The cohort of 1538 eligible adult participants included 769 who had AKI and a matched 769 who did not have AKI during their index hospitalization (Table 1). AKI during the index admission was predominantly stage 1 (15 % had stage 2 AKI, 13 % had stage 3 AKI, including 3 % who required acute renal replacement therapy but then had sufficient improvement in kidney function to stop dialysis). Mean follow-up was 4.6 (SD 1.9) years. During follow-up, 164 participants (82 from each group) withdrew from the study. Among those with AKI during the index hospitalization, median achieved SBP was 127 (interquartile range [IQR] 114–142) mmHg on a median of 2 (IQR 1–3) antihypertensive medications. Among those without AKI during the index hospitalization, median achieved SBP was a very similar 126 (IQR 114–137) mmHg on a median of 2 (IQR 1–3) antihypertensive medications.

**Table 1:** Baseline characteristics of ASSESS-AKI adult study population

Characteristic	AKI during index hospitalization (*n* = 769)	No AKI during index hospitalization (*n* = 769)	*P* value
**Measured at baseline visit**			
Systolic BP, mmHg, (mean, SD) (median, [IQR])	128.6 (22.0) 127 [114, 142]	126.6 (19.4) 126 [114, 137]	0.06
Diastolic BP, mmHg, (mean, SD) (median, [IQR])	71.3 (13.9) 71 [62, 80]	71.9 (13.8) 71 [62, 81]	0.39
Body mass index, kg/m^2^ (mean, SD)	31.6 (8.3)	30.5 (7.0)	0.007
Serum creatinine, mg/dL (mean, SD)	1.3 (0.7)	1.1 (0.4)	< 0.0001
CKD-EPI equation eGFR, ml/min/1.73 m^2^ (mean, SD)	65.7 (26.9)	72.7 (24.2)	< 0.0001
Urine protein to creatinine ratio, mg/gm (median, [IQR])	145.5 [81.1, 306.1]	117.6 [72.3, 222.2]	< 0.0001
Urine albumin to creatinine ratio, mg/gm (median, [IQR])	20.7 [8.0, 118.0]	11.3 [6.0, 32.4]	< 0.0001
**Medication use baseline study visit (n, %)**			
ACE-I/ARB	386 (50.2)	362 (47.1)	0.22
Diuretic	372 (48.4)	304 (39.5)	0.0005
Aldosterone receptor antagonist	65 (8.5)	53 (6.9)	0.25
Beta blocker	484 (62.9)	414 (53.8)	0.0003
Calcium channel blocker	191 (24.8)	166 (21.6)	0.13
Statin	451 (58.7)	430 (55.9)	0.28
Aspirin	94 (12.2)	89 (11.6)	0.69
Prescription NSAID	38 (4.9)	42 (5.5)	0.65
No. of BP medication classes at baseline study visit (mean, SD)*	2.2 [1.3]	1.8 [1.4]	< 0.0001
**Age (mean, SD)**	63.7 (12.8)	65.4 (12.6)	0.007
**Female (n, %)**	250 (32.5)	324 (42.1)	< 0.0001
**Center (n, %)**			
Kaiser Permanente	156 (20.3)	156 (20.3)	1
University of Washington	208 (27.1)	208 (27.1)	
Vanderbilt	251 (32.6)	251 (32.6)	
Yale consortium	154 (20.0)	154 (20.0)	
**Smoking status (n, %)**			
Never	308 (40.1)	326 (42.4)	0.31
Former	344 (44.7)	345 (44.9)	
Current	112 (14.6)	90 (11.7)	
Unknown	5 (0.7)	8 (1.0)	
**Race (n, %)**			
White	607 (78.9)	653 (84.9)	0.04
Black/African American	117 (15.2)	78 (10.1)	
Asian	17 (2.2)	14 (1.8)	
American Indian/Alaskan Native	9 (1.2)	5 (0.7)	
Native Hawaiian/Pacific Islander	4 (0.5)	6 (0.8)	
Multi-Racial	15 (2.0)	13 (1.7)	
**Hispanic (n, %)**	21 (2.7)	17 (2.2)	0.51
**Self-reported comorbidities (n, %)**			
Diabetes mellitus	387 (50.3)	271 (35.2)	< 0.0001
History of myocardial infarction or revascularization	372 (48.4)	321 (41.7)	0.03
Chronic kidney disease	306 (39.8)	306 (39.8)	1
Heart failure	205 (26.7)	122 (15.9)	< 0.0001
Hypertension	604 (78.5)	542 (70.5)	0.001
Chronic obstructive pulmonary disease	183 (23.8)	152 (19.8)	0.14
Chronic liver disease	38 (4.9)	22 (2.9)	0.07
Lupus	7 (0.9)	8 (1.0)	0.59
**Index hospitalization (n, %)**			
Treated in ICU	545 (70.9)	473 (61.5)	0.0001
Sepsis	118 (15.3)	26 (3.4)	< 0.0001
AKIN stage 1	553 (71.9)	NA	
AKIN stage 2	118 (15.3)		
AKIN stage 3	98 (12.7)		
Dialysis	26 (3.4)		

ACE-I, angiotensin converting enzyme inhibitor; ARB, angiotensin receptor blocker; AKI, acute kidney injury; AKIN, Acute Kidney Injury Network; eGFR, estimated glomerular filtration rate; ICU, Intensive care unit; CKD-EPI, Chronic Kidney Disease Epidemiology Collaboration

*Number of antihypertensive classes of medications included ACE-I/ARBs, renin inhibitors, alpha 2 agonists, alpha blockers, beta blockers, calcium channel blockers, vasodilators, anti-anginal (isosorbide derivatives), thiazide diuretics, loop diuretics, aldosterone receptor antagonists and potassium sparing diuretics at the baseline study visit

### Associations between SBP and outcomes of AKI, loss of kidney function, mortality and hospitalization for heart failure among the 769 patients with AKI during the index hospitalization

**Fig. 1:**
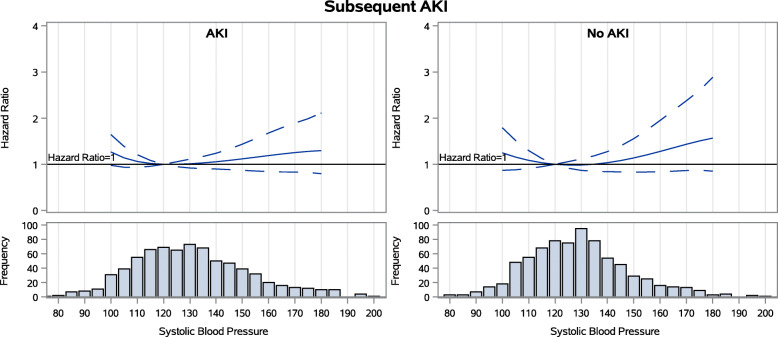
Spline models of the adjusted hazard ratios for subsequent hospitalized AKI by continuous systolic BP for patients with and without AKI during the index hospitalization

**Fig. 2:**
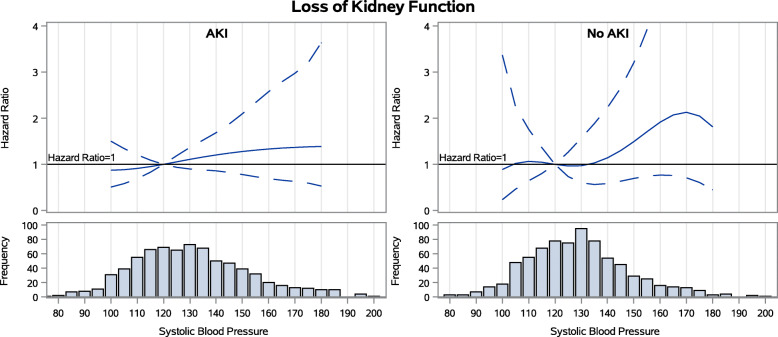
Spline models of the adjusted hazard ratios for loss of kidney function by continuous systolic BP for patients with and without AKI during the index hospitalization

**Fig. 3:**
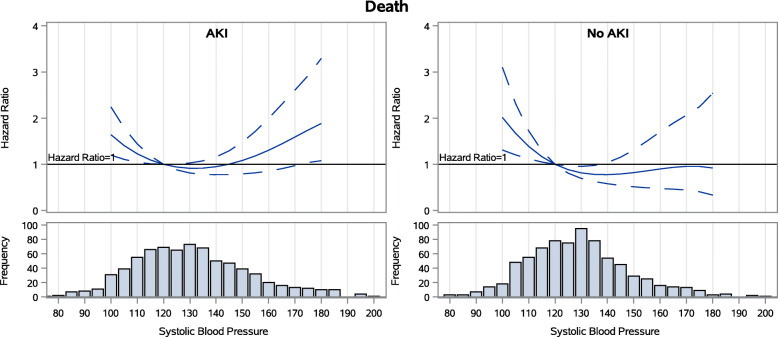
Spline models of the adjusted hazard ratios for all-cause mortality by continuous systolic BP for patients with and without AKI during the index hospitalization

**Fig. 4:**
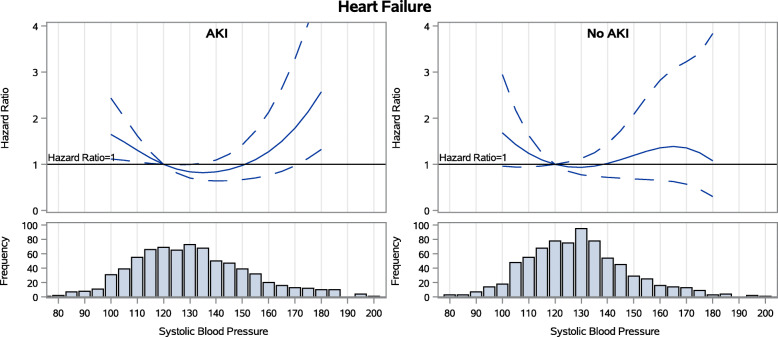
Spline models of the adjusted hazard ratios for heart failure hospitalization by continuous systolic BP for patients with and without AKI during the index hospitalization

During follow-up among the 769 patients with AKI during the index hospitalization, 42 % experienced subsequent AKI. 13 % experienced loss of kidney function (50 % drop in eGFR or ESKD) and 27 % died. 18 % had heart failure events. Achieved SBP 3 months after hospitalization did not have a stepwise association with any of the four adverse outcomes examined (Figs. 1, 2, 3 and 4)(unadjusted results are shown in Figures S2-S5). For subsequent AKI, the adjusted hazards of SBP 140 mmHg (compared to referent 120 mmHg) were 1.06 (95 % confidence interval [CI] 0.90–1.24)(Table S1). For loss of kidney function, the adjusted hazards were 1.20 (0.86–1.69). For all-cause mortality, the adjusted hazards were 0.95 (0.78–1.16). For heart failure hospitalization, the adjusted hazards were 0.84 (95 % confidence interval [CI] 0.64–1.09) in the AKI group.

### Associations between SBP and outcomes of AKI, loss of kidney function, mortality and hospitalization for heart failure among the 769 patients without AKI during the index hospitalization

During follow-up among the 769 patients without AKI during the index hospitalization, 26 % experienced subsequent AKI, 5 % experienced loss of kidney function, 15 % died, and 10 % had heart failure events. In this group, achieved SBP 3 months after hospitalization also did not have a stepwise association with any of the four adverse outcomes examined (Figs. 1, 2, 3 and 4). For subsequent AKI, the adjusted hazards of SBP 140 mmHg (compared to referent 120 mmHg) were 1.04 (0.84–1.28) (Table S1). For loss of kidney function, the adjusted hazards were 1.14 (0.58–2.23). For all-cause mortality, the adjusted hazards were 0.78 (0.58–1.04). For heart failure hospitalization, the adjusted hazards were 1.02 (0.72–1.45).

### Assessment for interaction by AKI in the full cohort

Graphs of the adjusted hazard ratios for each outcome by continuous SBP were similar in patients with and without AKI during the index hospitalization (Figs. 1, 2, 3 and 4). When the full cohort of all 1538 patients with and without AKI during the index hospitalization were combined in the same model, AKI did not modify the associations between post-hospitalization SBP and subsequent AKI (*p* = 0.27), loss of kidney function (*p* = 0.26), mortality (*p* = 0.55), or heart failure events (*p* = 0.52).

### Diastolic Blood Pressure

In adjusted analysis, achieved DBP 3 months post-hospitalization also had no stepwise association with of any of the four adverse outcomes examined (Supplemental Figures S6-S9). We observed no modification by index AKI status on any of these associations.

### Sensitivity analyses

When we repeated the analysis using alternative subsequent AKI definitions of (1) a 50 % relative increase or (2) either a 0.3 mg/dL absolute increase or 50 % relative increase from the most recent outpatient SCr to the peak SCr during a hospitalization, the incidences of AKI were 24 and 34 % respectively (compared with 34 % with the original definition). Similar to the main analysis, lower achieved SBP 3-month post-hospitalization was not associated with stepwise higher risk of subsequent AKI (Supplemental Figures S10-S11). No effect modification by index hospitalization AKI was found.

## Discussion

In this prospective observational study of ASSESS-AKI participants, in contrast to our first *a priori* hypothesis, we found that achieved SBP 3 months after hospitalization did not have a stepwise association with any of the four adverse outcomes examined among AKI survivors. This was true in particular within the range of SBP 110–150 mmHg, where targets for interventional trials would almost certainly be set. Associations between 3-month post-hospitalization SBP and subsequent risks of hospitalized AKI, loss of kidney function, all-cause mortality, and adjudicated heart failure events were similar in participants with and without AKI during the index hospitalization, confirming our second *a priori* hypothesis.

Our results are not consistent with what one would expect based on the landmark SPRINT trial, which found that targeting a SBP of < 120 mmHg lowered all-cause mortality and major cardiovascular events compared with targeting a SBP of < 140 mmHg [19]. In contrast, we did not find that ASSESS-AKI study participants with SBP 140 mmHg had higher rates of adverse events than their counterparts with SBP 120 mmHg. This may be due to differences in patient population. SPRINT enrolled ambulatory patients without diabetes mellitus but with elevated cardiovascular risk, whereas all ASSESS-AKI enrollees were recently hospitalized. SPRINT patients had a mean SBP of 140 mmHg at baseline, whereas ASSESS-AKI enrollees had baseline mean SBP < 130 mmHg. It is possible that recent hospitalization alters the association between SBP and adverse outcomes. Another possibility is that our observational data are confounded by unmeasured differences among patients achieving different blood pressures. For instance, we were not able to control for low ejection fraction in this analysis, which may result in low blood pressure without antihypertensive medication. It is less likely that we missed true stepwise associations due to being underpowered, given the shapes of the splines and widths of the confidence intervals in the SBP region of interest. Further studies are needed to see if these observations can be replicated in other cohorts of survivors of hospitalized AKI [20].

An important strength of this analysis is that it is the first and only one to our knowledge to explore how observed blood pressure measured systematically after discharge from hospitalizations complicated by AKI relates to subsequent adverse outcomes. To strengthen the rigor of our study, we analyzed information collected systematically as part of a structured research protocol, rather than relying on data collected as part of routine clinical care, thus reducing potential ascertainment bias and missing data. We evaluated three definitions of subsequent AKI and results were consistent across all definitions. We were able to systematically account for key covariates such as amount of proteinuria and eGFR—both quantified at a uniform time point relative to BP ascertainment and to the AKI episode for better control of confounding. We also had long-term follow-up for clinical outcomes (mean follow-up 4.6 years), comparable with large trials of BP targets. The ability to compare the association between BP and adverse outcomes in those who did and did not experience AKI during the index admission is another novel aspect of this work.

Our study had several limitations. First, this was an observational study of achieved BP without any defined targets. Thus, in some instances, we compared outcomes of patients who were treated with antihypertensive medications to achieve a certain blood pressure with outcomes of patients who achieved the same blood pressure without medication (although 84 % of the cohort was on antihypertensive medication). Although our model includes antihypertensive medication classes, we did not account for dosing differences and residual confounding is possible. We defined BP at a single point in time (the 3-month post-hospitalization baseline study visit) and BP may have changed during follow-up. However, this approach has been commonly used in observational studies associating BP with clinical outcomes [21–23]. While we had few patients with SBP > 160 mmHg or < 100 mmHg, management of SBP in these ranges is not controversial and no contemporary clinical trial would pick a SBP target of > 160 mmHg or < 100 mmHg. Since the majority of AKI episodes among ASSESS-AKI enrollees were stages 1 and 2, our findings may or may not apply to survivors of more severe AKI. AKI etiology was not available, and AKI of different etiologies (e.g. pre-renal azotemia versus acute tubular necrosis) may have differential effects on associations between SBP and outcomes.

## Conclusions

In conclusion, in this prospective cohort study, AKI (predominantly stage 1 AKI) was not an effect modifier of the associations between BP and adverse outcomes after hospitalization. Furthermore, there was not a strong stepwise association between BP and subsequent adverse outcomes among AKI survivors.

The opinions expressed in this article are the authors’ own and do not reflect the view of the National Institute of Diabetes and Digestive and Kidney Diseases, the National Institutes of Health, the Department of Health and Human Services, or the United States Government.

## Supplementary information

**Additional file 1.**

## Data Availability

The data analyzed in the current study are available in the NIDDK Central Repository: https://repository.niddk.nih.gov/studies/assess-aki/.

## Abbreviations

AKI: acute kidney injury
BP: blood pressure
CKD: chronic kidney disease
eGFR: estimated glomerular filtration rate
ESKD: endstage kidney disease
SBP: systolic blood pressure
SCr: serum creatinine concentration
