# Supplementary material for: Achieved blood pressure post-acute kidney injury and risk of adverse outcomes after AKI: A prospective parallel cohort study
Source: BMC Nephrol. 2021 Jul 29;22:270. doi: 10.1186/s12882-021-02480-1 (PMC8320241; doi:10.1186/s12882-021-02480-1)
Supplement: Supplementary file 1 — Additional file 1. [file 12882_2021_2480_MOESM1_ESM.docx]

**SUPPLEMENTAL MATERIAL**

Figure S1. Cohort assembly


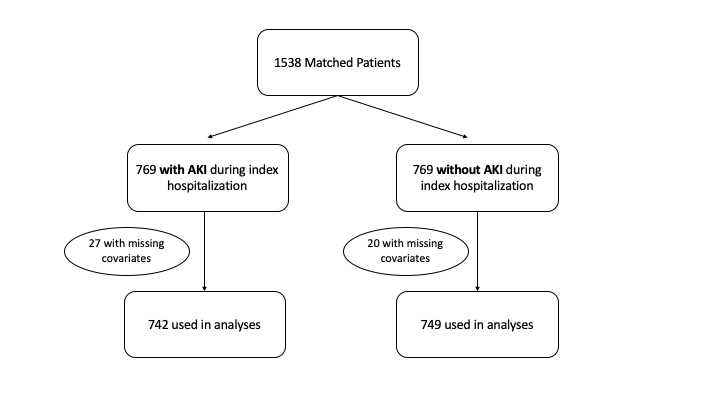


**Unadjusted primary analyses:**

Figure S2. Spline models of the unadjusted hazard ratios for subsequent hospitalized AKI by continuous systolic BP for patients with and without AKI at the index hospitalization.


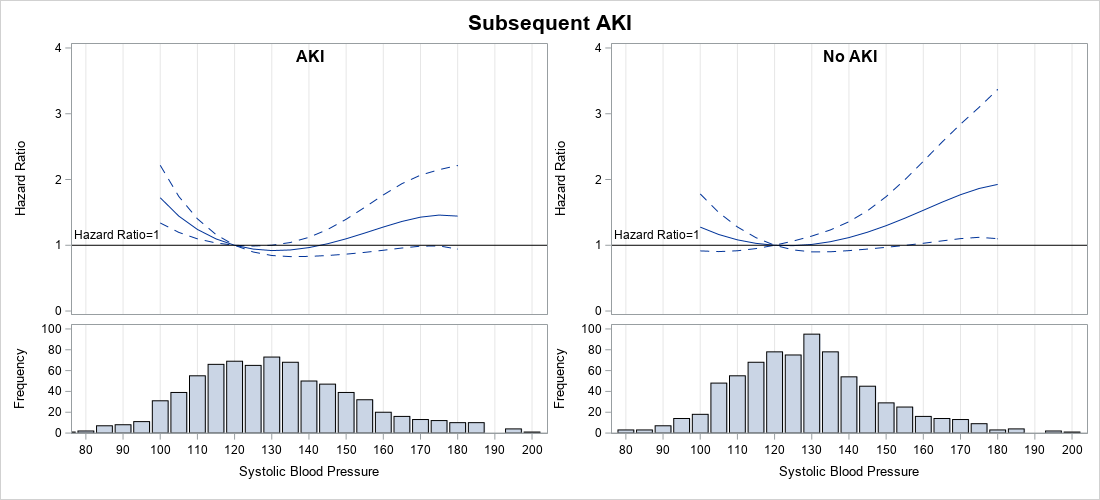


Figure S3. Spline models of the unadjusted hazard ratios for loss of kidney function by continuous systolic BP for patients with and without AKI at the index hospitalization.


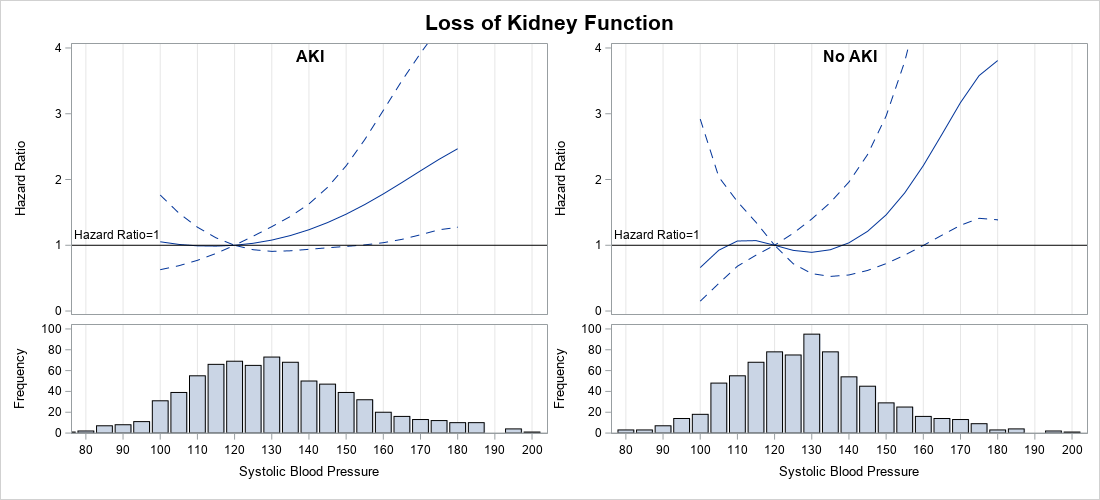


Figure S4. Spline models of the unadjusted hazard ratios for mortality by continuous systolic BP for patients with and without AKI at the index hospitalization.


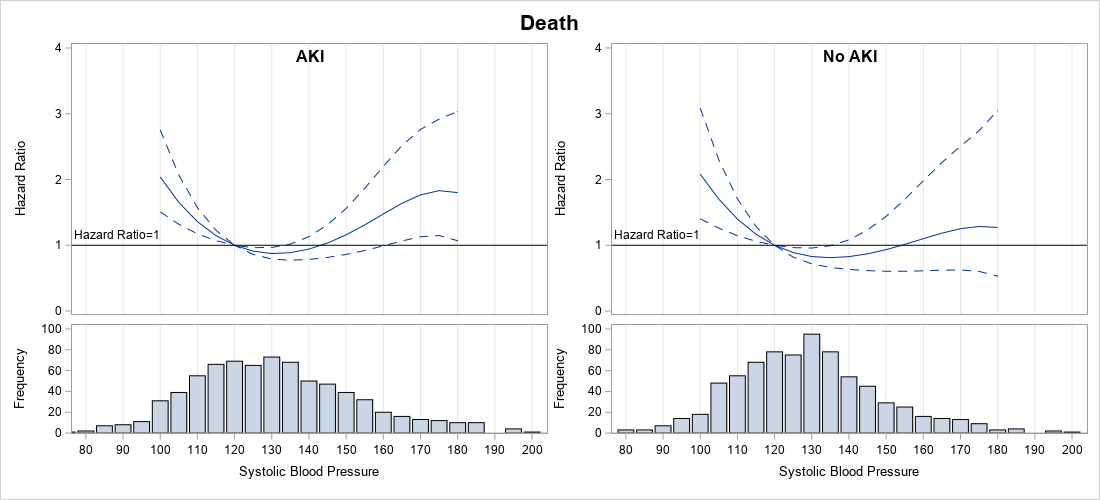


Figure S5. Spline models of the unadjusted hazard ratios for heart failure hospitalizations by continuous systolic BP for patients with and without AKI at the index hospitalization.


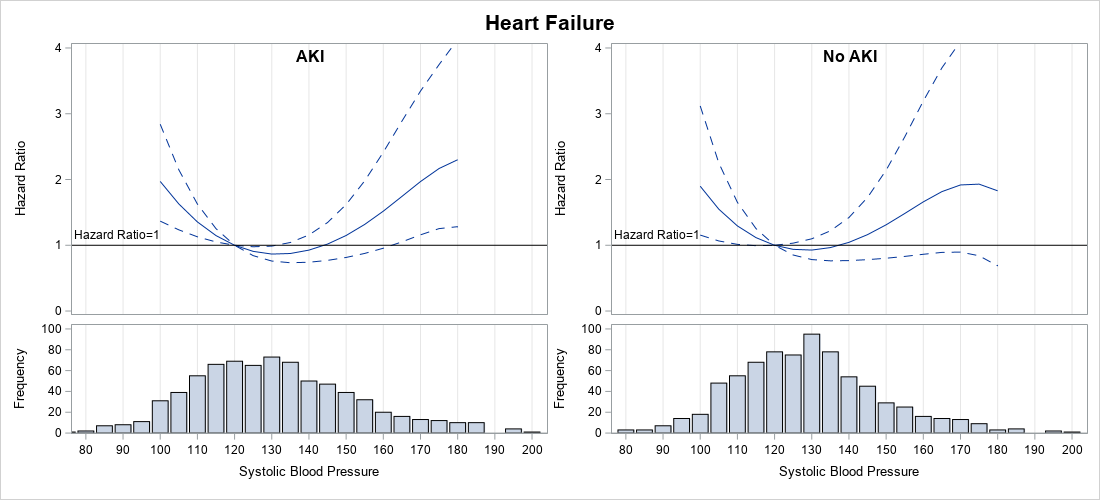


**Diastolic BP Analyses:**

Figure S6. Spline models of the adjusted hazard ratios for subsequent AKI by continuous diastolic BP for patients with and without AKI at the index hospitalization.


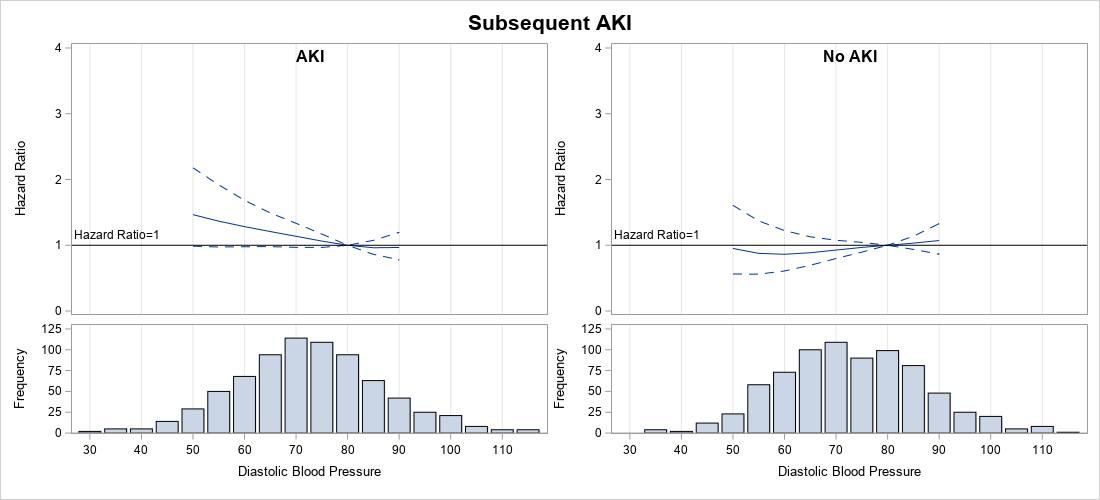


Figure S7. Spline models of the adjusted hazard ratios for loss of kidney function by continuous diastolic BP for patients with and without AKI at the index hospitalization.


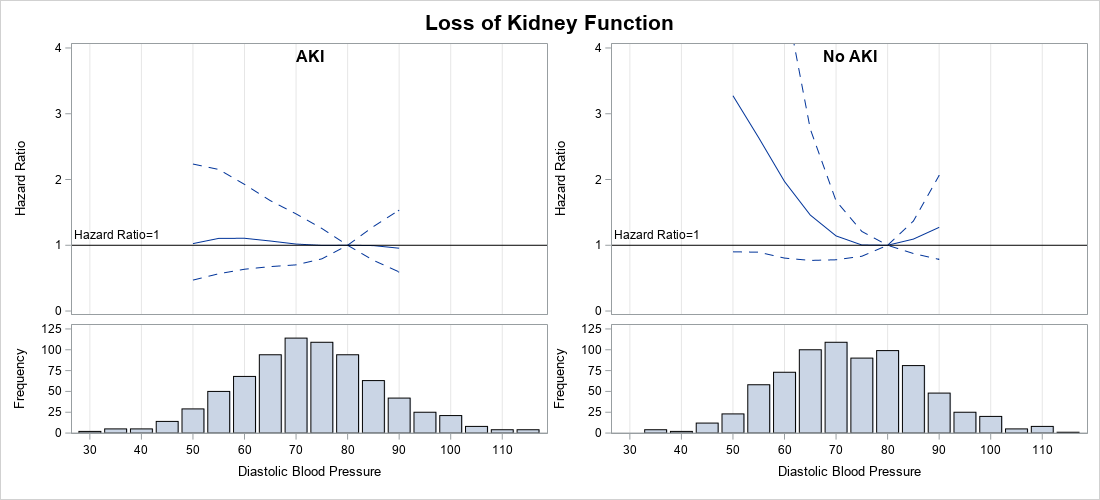


Figure S8. Spline models of the adjusted hazard ratios for mortality by continuous diastolic BP for patients with and without AKI at the index hospitalization.


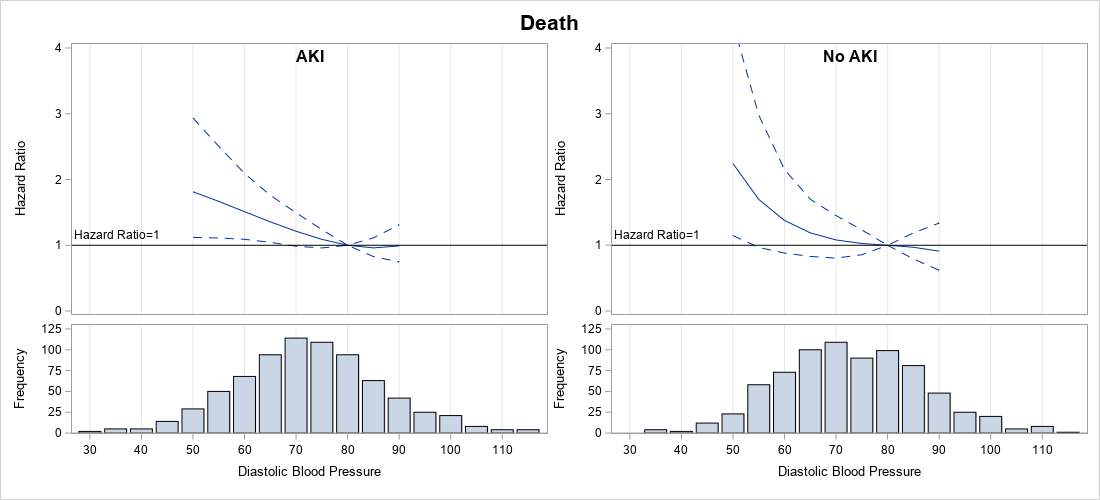


Figure S9. Spline models of the adjusted hazard ratios for heart failure hospitalizations by continuous diastolic BP for patients with and without AKI at the index hospitalization.


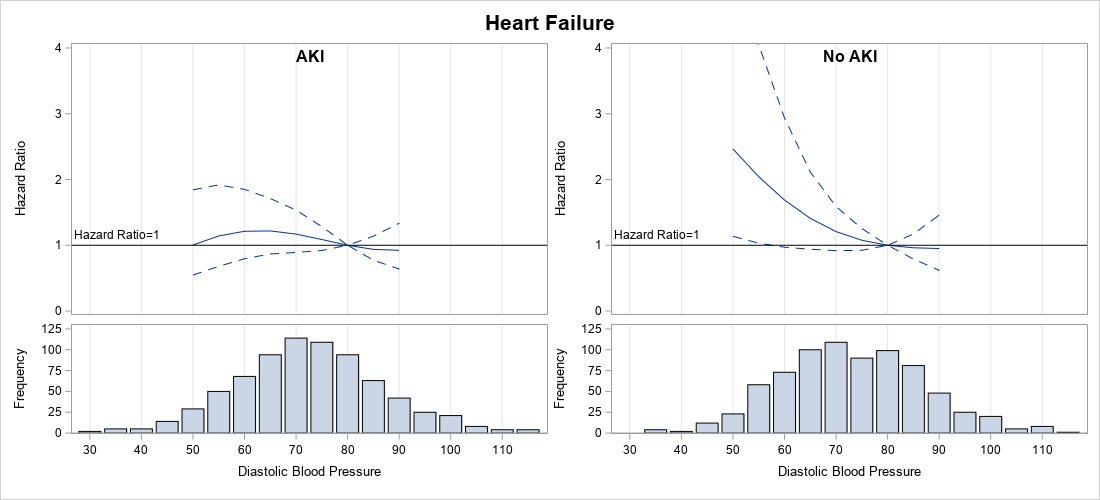


**Sensitivity Analyses for Subsequent AKI:**

Figure S10. Sensitivity analysis with AKI defined as a >50% relative increase from the most recent outpatient study visit SCr to the peak inpatient SCr: Spline models of the adjusted hazard ratios for subsequent AKI by continuous systolic BP for patients with and without AKI at the index hospitalization.


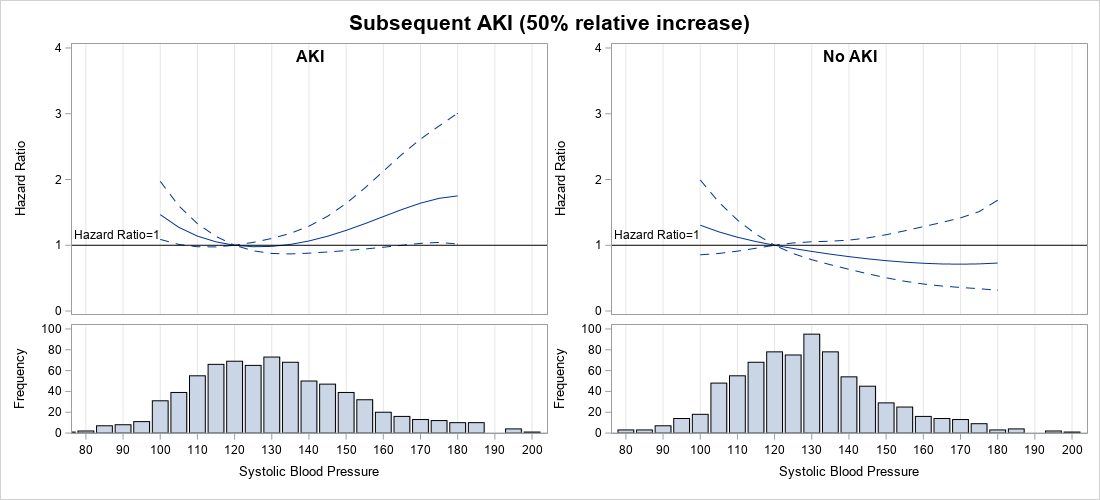


Figure S11. Sensitivity analysis with AKI defined as a >50% relative increase or ≥0.3 mg/dL absolute increase from the most recent outpatient study visit SCr to the peak inpatient SCr: Spline models of the adjusted hazard ratios for subsequent AKI by continuous systolic BP for patients with and without AKI at the index hospitalization.


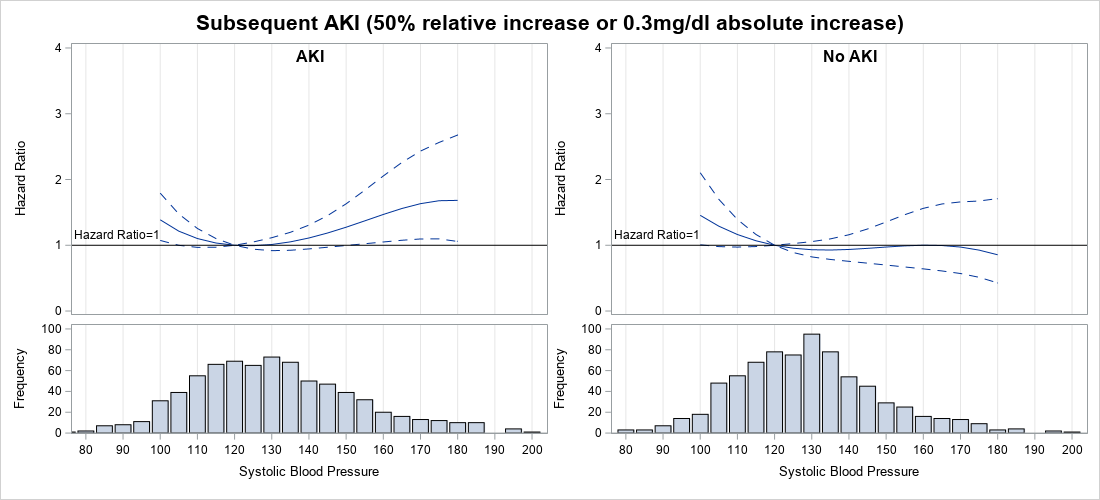


Table S1. Adjusted hazard ratios for SBP 140 mmHg (referent 120 mmHg).

| Adjusted models | | | |  |
| --- | --- | --- | --- | --- |
|  | Hazard of SBP of 140 (compared to referent 120) in AKI group | Hazard of SBP of 140 (compared to referent 120) in group without AKI | Ratio of hazards | P value for ratio of hazards |
| Subsequent AKI | 1.055  (0.898, 1.240) | 1.035  (0.840, 1.276) | 1.019  (0.783, 1.327) | 0.888 |
| Loss of kidney function | 1.203  (0.859, 1.686) | 1.142  (0.584, 2.234) | 1.054  (0.497, 2.234) | 0.891 |
| All-cause mortality | 0.948  (0.775, 1.160) | 0.776  (0.579, 1.040) | 1.222  (0.856, 1.745) | 0.269 |
| Heart failure events | 0.837  (0.640, 1.094) | 1.020  (0.719, 1.448) | 0.820  (0.528, 1.274) | 0.378 |
